# "Boom" and "Bust" cycles in virus growth suggest multiple selective forces in influenza a evolution

**DOI:** 10.1186/1743-422X-8-180

**Published:** 2011-04-18

**Authors:** Rajagowthamee R Thangavel, Aisha Reed, Erin W Norcross, Sherrina N Dixon, Mary E Marquart, Stephen J Stray

**Affiliations:** 1 Department of Microbiology, University of Mississippi Medical Center, 2500 N State St, Jackson, MS 39216, USA

## Abstract

**Background:**

Influenza A virus evolution in humans is driven at least in part by mutations allowing the virus to escape antibody neutralization. Little is known about the evolution of influenza in birds, a major reservoir of influenza A.

**Methods:**

Neutralizing polyclonal antiserum was raised in chicken against reassortant influenza virus, CalX, bearing the hemagglutinin (HA) and neuraminidase (NA) of A/California/7/2004 [H3N2]. CalX was serially passaged in the presence of anti-CalX polyclonal IgY to derive viruses capable of growth in the presence of antibody.

**Results:**

Polyclonal chicken antibody neutralized both HA activity and infection by CalX, but had no effect on a strain bearing an earlier human H3 and an irrelevant neuraminidase (A/Memphis/71-Bellamy/42 [H3N1]). Surprisingly, most of the antibody-resistant viruses were still at least partially sensitive to neutralization of HA activity and viral infection. Although mutant HA genes bearing changes that might affect antibody neutralization were identified, the vast majority of HA sequences obtained were identical to wild type, and no individual mutant sequence was found in more than one passage, suggesting that those mutations that were observed did not confer sufficient selective advantage to come to dominate the population. Different passages yielded infectious foci of varying size and plaques of varying size and morphology. Yields of infectious virus and relative frequency of different morphologies changed markedly from passage to passage. Sequences of bulk, uncloned PCR products from antibody-resistant passages indicated changes in the PB2 and PA proteins with respect to the wild type virus.

**Conclusions:**

Each antibody-selected passage consisted of a variety of different cocirculating populations, rather than pure populations of virus able to escape antibody by changes in antibody epitopes. The ability to escape antibody is apparently due to changes in genes encoding the viral polymerase complex, probably resulting in more robust viral replication, allowing the few virus particles not completely neutralized by antibody to rapidly produce large numbers of progeny. Our data suggest that the relative success of an individual variant may depend on both its own gain and loss of fitness, as well as that of its cocirculating variants.

## Background

Influenza A virus causes recurrent seasonal epidemics, and pandemics that occur every few decades. All known antigenic subtypes can be isolated from avian species, especially waterfowl [1], indicating that birds are particularly important reservoirs of influenza A virus diversity. Two major mechanisms are known to drive evolution of influenza A viruses in humans: antigenic shift, when one or more of the eight viral gene segments is exchanged by reassortment between influenza virus isolates [1,2], and antigenic drift, where mutations accumulate in viral genes, especially those encoding the surface antigens hemagglutinin (HA) and neuraminidase (NA) [3,4]. Both processes reduce the effectiveness of pre-existing immunity in the host by ablating epitopes recognized by antibodies, and, to a lesser extent, T-cells [5].

Influenza A virus strains isolated from successive seasonal influenza epidemics typically differ by a very small number of amino acids in HA and NA. For some antibody/antigen combinations, it has been shown that the majority of the binding energy is contributed by interactions with a single amino acid within the epitope [6,7], so changing a single critical amino acid might constitute a "jackpot" solution to the problem of antibody neutralization. Although theoretically the human antibody response is almost infinitely diverse, the repertoire of anti-influenza antibodies within an individual is apparently quite restricted [8].

Since the primary antibody repertoire of birds is inherently less diverse than in humans [9], it may be that a very limited number of specificities would occur in individual birds. Thus, point mutations in the surface antigens, particularly HA, might confer significant competitive advantage for the virus in the presence of antibody. Other properties, such as increased affinity for cellular receptors [10], alterations in fusion pH [11], increased RNA replication or transcription [11], or increase in the yield of viral particles from each infected cell (burst size) may all act to enhance competitiveness. Recent studies of human seasonal influenza demonstrated both significant founder effects which may complicate the understanding of the role of individual mutations [12], and changes in replication genes leading to enhanced competitiveness, allowing complete replacement of one major circulating strain by another [2], strongly suggesting that antibody escape alone is not the only means whereby one virus variant can achieve dominance over others in the same population.

Given that influenza viruses of avian origin can cause fatal epizootic infections of humans, and influenza viruses of birds are an important precursor to human pandemics, we wished to examine the role, if any, of polyclonal antibody from an avian species in an *in vitro *model of influenza A virus evolution. We have chosen to use chicken (*Gallus gallus domesticus*) as a model system, since chicken antibody genetics and function is probably the best studied of all avian species, and polyclonal antibodies can be generated and purified with relative ease [13]. Chicken polyclonal antibodies were raised against a recent reassortant vaccine strain carrying the hemagglutinin (HA) and neuraminidase derived from A/California/7/2004 (H3N2) and internal genes from a standard laboratory strain, A/Puerto Rico/8/34 (H1N1 [PR8]). Since this strain is only distantly related at the antigenic level to any strain currently circulating in birds, it is likely to be highly antigenic in chickens, and the use of a H3N2 strain would allow direct comparison of any chicken epitope identified with epitopes of human and murine antibodies that have been characterized at the structural level.

We obtained antiserum that both neutralized HA activity *in vitro*, and inhibited virus growth in tissue culture. We were able to select virus populations that were enhanced for their ability to grow in the presence of polyclonal antiserum. Curiously, although we were able to detect individual HA molecules within these populations having mutations in or near previously described neutralizing epitopes, these mutant HA sequences did not take over the populations at any point, despite many rounds of selection in the presence of antiserum. Instead, we observed transient increases in plaque size in some populations, suggesting that viruses in these populations replicated faster or produced more progeny than the parental virus.

Our data suggest that avian antibodies can select mutant viruses with changes in the HA molecule, but that changes in viral replication apparently played a greater role in shaping the viral repertoire. A better understanding of the evolutionary process may allow us to improve vaccine design to combat future influenza pandemics.

## Methods

### Cells and Viruses

Madin-Darby canine kidney (MDCK) cells (ATCC CCL-34) were cultured in MDCK growth medium (DMEM/Ham's F12 1:1 [Mediatech]), supplemented with 10% fetal bovine serum, 50 U/ml penicillin and 50 μg/ml streptomycin [all Invitrogen]). Influenza A viruses used in this study were A/reassortant/California/7/2004xPR8, H3N2, CDC#2005712034 [CalX], a reassortant virus bearing the HA and NA from A/California/7/2004, and A/Memphis/1/71-Bellamy/42 (H3N1) [Mem-Bel] [14]. Both were kind gifts of Gillian M. Air (Oklahoma City, OK). Virus stocks were grown in MDCK cells under limiting dilution, and harvested at 96 h post infection (p.i.). Influenza-infected MDCK cells were cultured in infection medium (DMEM:Ham’s F12 1:1 [Mediatech]), supplemented with 1% ITS+ [Becton Dickinson], 50 U/ml penicillin and 50 μg/ml streptomycin [both Invitrogen], and 0.5 μg/ml TPCK-trypsin [Worthington]) as previously described [15]. Both CalX and Mem-Bel have been passaged extensively in MDCK cells, and were sequentially passaged in MDCK cells at least five times in our hands prior to initiation of these studies to ensure that they were completely adapted to our culture conditions.

### Antisera

Rabbit polyclonal antiserum was raised under supervision of the University of Mississippi Medical Center Institutional Animal Care and Use Committee against NWS-G70c "cores" disrupted by sonication [16]. Specificity of this antiserum was tested by western blot demonstrating recognition of both NP and M proteins. Polyclonal chicken antiserum (isotype IgY) against CalX was raised commercially using whole, sucrose gradient purified egg-grown virus disrupted by sonication as antigen. Both preimmune and immune polyclonal IgY were purified from egg yolk (Gallus Immunotech, Fergus, Ontario). We chose to use a human-adapted virus out of concern that certain epitopes might be immunodominant in avian species, and thus an avian-adapted virus might lack critical epitopes highly antigenic in chickens. Note that human H3N2 viruses have been extensively characterized at the structural and antigenic level.

### HA and HAI assays

HA assays were performed as described [17]. Human RBC were obtained as de-identified, discarded, diagnostic specimens from the G.V. "Sonny" Montgomery VA Medical Center, Jackson MS, with Institutional Review Board approval. HAI was performed by serially diluting antibody two-fold into wells containing 8 HAU of virus. The HAI titer is the reciprocal of the highest dilution showing complete inhibition of HA.

### Generation of chicken polyclonal IgY resistant CalX passages

We determined the minimal antibody concentration required to inhibit infection of MDCK cells by our virus stocks by infecting at a multiplicity of infection (m.o.i.) of approximately 0.01 infectious units per cell in the presence of antibody concentrations ranging from 500 to 0.005 HAIU/ml (10^-2 ^to 10^-7 ^dilutions of stock IgY). Infection was completely inhibited by 500 HAIU/ml IgY (10^-2 ^dilution of stock), and was barely detectable when 50 HAIU/ml IgY (10^-3 ^dilution of stock). To generate antibody-resistant viruses, infections were performed in the presence of antibody by diluting virus directly into 50 HAIU/ml chicken polyclonal IgY (10^-3 ^dilution of stock IgY) in six-well plates containing MDCK cells. Virus and antibody were incubated with MDCK cells at 37°C for 1 h to allow virus adsorption to cells, followed by addition of infection medium containing 0.5 μg/ml TPCK-trypsin. The initial inoculum was not removed, and further antibody was not added to the infection medium. Although this was done to conserve the limited supply of IgY, we feel this also models the effect of antibody in the host, which is most likely to exert its effect at the stage of the initial exposure. Culture supernatants were collected from last well positive for HA at approximately 96 h p.i., and were sequentially passaged at limiting dilution in the presence of the same amount of antibody. Thus, each round of selection began with 1-9 particles which were infectious in the presence of antibody, and the progeny virus were the result of up to eight rounds of infection [18].

### Calculation of burst size (particles released per infected cell)

Burst size calculations were performed as described previously [18]. Briefly, it was assumed that all the progeny virus detected from a well where cells were completely lysed was produced in the final round of infection, and that cells in this well were confluent prior to this final infectious cycle. We have previously determined that a confluent monolayer in a 16 mm well is equivalent to 2 × 10^5 ^cells [11], and one HA unit is equivalent to 10^6 ^particles [18]. It should be noted that, since burst size calculations are based on HA, differences not greater than two-fold are probably not significant. While this calculation is somewhat crude, it should be internally consistent and thus allow useful comparisons of progeny virus from passage to passage.

### Plaque assay

Plaque assays were performed as previously described [19,20]; virus was adsorbed to MDCK cells seeded in 6-well plates as described above, washed with PBS and overlayed with 0.9% caboxymethylcellulose (CMC) in DMEM (high glucose, Thermo Scientific) supplemented with 0.5 μg/ml TPCK-trypsin, but containing no serum or ITS+. The wells were simultaneously fixed and stained at 72 hrs post infection with 0.25% crystal violet in 20% formalin/40% ethanol. Where plaque assays were carried out in the presence of IgY, antiserum was diluted 10^-3 ^fold and present only during virus adsorption.

### Focus formation assay

MDCK cells were seeded on ethanol-washed glass cover slips 16-24 h prior to infection. Cells were infected as described for plaque assays, except that cells were overlayed with CMC in infection medium plus 0.5 μg/ml TPCK-trypsin. At 48 h p.i., cover slips were fixed with 3% paraformaldehyde in PBS for 12 to 16 hrs, permeabilized with ice-cold methanol for 2 min, then stained with anti-NWS-G70c rabbit primary antibody and FITC-conjugated sheep anti-rabbit IgG (Sigma Aldrich). The number of fluorescent cells per infected focus was counted by fluorescence microscopy and subjected to statistical analysis either by the Mann-Whitney non-parametric *U *test or Kruskal-Wallis one-way analysis of variation using Prism 4.0 (GraphPad Software, La Jolla, CA).

### RNA Isolation, cDNA cloning, and sequencing

Viral RNA was isolated using RNeasy Mini Kit, (Qiagen) and reverse transcribed using Superscript III [Invitrogen], using a modified influenza A universal primer (primer 2, see Additional file 1, Table S1). The cDNA was amplified by PCR with either *Pfx*50 high-fidelity DNA polymerase or *Taq *DNA polymerase (Invitrogen) as indicated, using primer pairs specific for the H3 HA gene ([21,22], Additional file 1, Table S1 primers 20 and 21 or 23 and 39). Amplified products were purified using MinElute Gel Extraction kits (Qiagen), then cloned using either Zero Blunt TOPO (for *Pfx*50-generated products, Invitrogen) or, for *Taq*-generated products, by ligating into pCR^®^2.1 (Original TA Cloning Kit, Invitrogen) according to manufacturer's instructions. Plasmid DNA was prepared using QIAprep minispin DNA kit, and screened for inserts by restriction digestion. Positive clones were sequenced (Laragen, Culver City, CA) using either the forward or reverse primer used for PCR amplification. Sequences were compared to those in publicly available databases using the Basic Local Alignment Search Tool (BLAST, http://www.ncbi.nlm.nih.gov/BLAST/).

### Sequencing of uncloned PCR products

Viral RNA was isolated and reverse transcribed as above, except that cDNA synthesis was primed using either the primer 1 or primer 2 (Additional file 1, Table S1). PCR products were generated using gene segment specific primers (Additional file 1, Table S1). Full-length products were not generated efficiently for PB1, PB2, and PA, so these were amplified as 5' and 3' gene fragments generated using a combination of terminal and internal gene-specific primers (Additional file 1, Table S1). PCR products were purified by from agarose gels using Qiagen MinElute gel extraction kit, and sequenced using either the M13 forward or reverse primer, or the same primer used for PCR amplification (Laragen Inc, Culver City CA). To unequivocally determine whether any changes had occurred at the extreme 5' and 3' ends, these were sequenced using gene-specific reverse primers near the 5'end and forward primers near the 3'ends, respectively (Additional file 1, Table S1). Accession numbers for all sequences are shown in Additional file 1, Table S2.

## Results and Discussion

### Chicken IgY inhibits both hemagglutination and infection

To test whether avian antibodies can neutralize influenza virus, and thus have the potential to select novel influenza viruses in birds, we raised polyclonal antiserum in domestic chicken (*Gallus gallus domesticus*). We tested IgY purified from eggs of a chicken immunized with CalX, a reassortant virus bearing the HA and NA from A/California/7/2004, for its ability to inhibit hemagglutination activity of CalX and Mem-Bel, a reassortant virus comprising an earlier human H3 HA and irrelevant NA [14]. The IgY preparation strongly inhibited hemagglutination by CalX (HA inhibitory [HAI] titer 51200 HAIU/ml) but had minimal cross-reactivity to Mem-Bel (HAI <20 HAIU/ml). We determined that antibody concentrations of 500 HAIU/ml and higher completely inhibited infection of MDCK cells by our virus stocks, while 50 HAIU/ml of antiserum allowed minimal outgrowth of wild-type CalX. No virus outgrowth was observed in the presence of 500 HAIU/ml antibody, even when supernatant was passaged in the absence of antibody to amplify any virus present but below the level of detection. Infections performed at lower antibody concentration were indistinguishable from those performed in the absence of antibody. Infection by CalX was at least 10^4^-fold inhibited when cells were infected in the presence of 50 HAIU/ml IgY, whereas the growth of Mem-Bel was inhibited less than 10-fold. The polyclonal anti-CalX IgY can therefore neutralize both hemagglutination and infection, is specific for CalX, and does not recognize neutralizing sites in the older Memphis/1/71 HA. Thus, some antibodies bind sufficiently tightly to the CalX HA molecule to block its function, and at least some of these binding sites are probably similar to human epitopes since Mem/71 and CalX HA molecules differ most in these areas.

### Infection in the presence of IgY produces antibody-resistant isolates

Virus grown by infecting in the presence of IgY was further passaged by infecting fresh cells in the presence of IgY with 10-fold serial dilutions of progeny virus from the well corresponding to the highest dilution of inoculum showing virus outgrowth. Thus, for every round of infection, the inoculum used represented virus derived from infection at the highest ratio of antibody to virus, where the effect of antibody selection should be strongest. Because these viruses selected in the presence of antibody infect more efficiently in the presence of antibody than does wild-type, it must be assumed that they have acquired some phenotype which confers a selective advantage in the presence of IgY.

### IgY-selected isolates display a cyclic "boom" and "bust" pattern of virus yield and burst size

**Figure 1:**
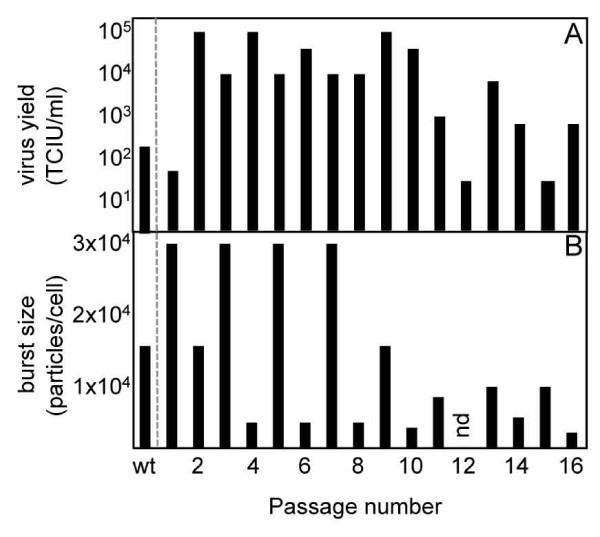
**"Boom" and "bust" cycles in influenza virus under serial antibody selection**. Influenza A virus CalX was serially cultured in the presence of neutralizing chicken polyclonal IgY antiserum raised against the same virus. Infectivity in the presence of antibody (A), and burst size (B) were determined for each passage (nd: no data available).

Yield of virus infectious in the presence of IgY in each passage increased from fewer than 100 tissue-culture infectious units per milliliter (TCIU/ml) after the first round of selection to 10^5 ^TCIU/ml (Figure 1). The yield displayed a cyclic "boom" and "bust" pattern over the course of the study. A downward trend in yield ("bust") was apparent after passage 9, and reached titers similar to the titer at the first round of selection at passage 12. Yield increased 100 fold between passages 12 and 13 ("boom"), but declined again in passages 14 and 15, recovering again in passage 16. Passage 2 had 1000-fold higher yield than the first round of selection. For simplicity, we will refer to passages 1-10 as "early" passages, while passages 11 and higher will be designated "late". Note that a second series of passages derived in parallel showed a similar pattern, but the yield of this series could not be detected after the ninth passage. Burst size, the number of particles produced per infected cell, was calculated [11,18]. Burst size also varied with passage number (Figure 1). Burst size values ranged from 1.9 × 10^3 ^particles/cell (passage 16) to 2.6 × 10^4 ^particles/cell (passages 1, 3, 5, and 7). Burst sizes and yield of infectious virus were generally lower in the later passages than in the earlier passages, suggesting that, on average, infected cells produced more progeny during the earlier phase of the selection process than in later passages. Note that, since yields of infectious virus were measured in the presence of IgY, we would not necessarily expect the infectious yields and burst sizes to correlate, since the burst size calculations include all particles irrespective of their ability to infect in the presence of antibody.

The decrease both in infectious yield and burst size suggests that some passages have lost fitness, at least under antibody selection. A possible explanation is that passages with reduced fitness contain a high proportion of defective interfering (DI) particles. Influenza DI particles have been demonstrated in virus grown at high multiplicity of infection [23]. Influenza DI particles interfere with replication of homologous viruses (i.e., those from which they are derived) [24], although heterosubtypic interference has been reported [25]. The ability of influenza DI's (or even different strains) to interfere with replication *in vitro *is cell-line dependent in at least some cases [25,26]. While we do see an increase in particle to focus formation ratio in some strains (see below), which might indicate the presence of DI particles, all of our strains are derived by infection at limiting dilution (i.e., lowest possible effective m.o.i.), which is inconsistent with production of DI particles in previous studies. We also note that single-plaque passaging studies on vesicular stomatitis virus (VSV) yielded individual lineages that showed consistent increases or decreases in fitness over the course of twenty cycles of selection, but the boom and bust dynamics of fitness seen in our studies was not observed for VSV [27]. Due to the potential for reassortment of influenza virus gene segments during mixed infection, direct fitness comparisons between virus strains cannot be performed for our viruses as for VSV.

### IgY-selected viruses remain sensitive to neutralization by antibody

**Figure 2:**
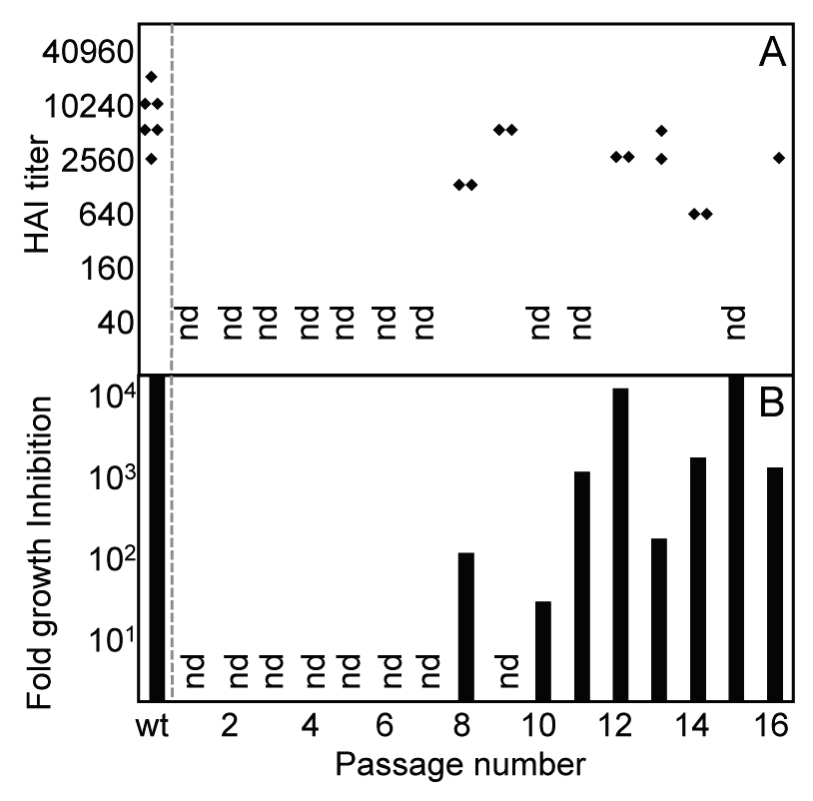
**Successful growth in the presence of IgY does not correlate with lack of susceptibility to neutralization**. Selected passages were tested for sensitivity to hemagglutination inhibition (HAI) by immune IgY (A), and neutralization of infection compared to infection in the absence of IgY (B). Note that the y-axis in panel *B *is logarithmic (nd: no data available).

To determine whether the IgY-selected passages were resistant to antibody neutralization, we infected MDCK cells with selected passages in parallel with or without IgY. Neutralization-resistant viruses will be equally infectious in the presence and absence of antibody, whereas those which are inhibited by IgY will have a lower apparent infectious titer in the presence of antibody. Surprisingly, we found all passages tested to be at least partially inhibited by IgY at the level of infection. The anti-CalX IgY does not inhibit infection by Mem-Bel at these levels, so the inhibition of virus infection cannot be due to some indirect effect such as antibody blocking cell surface receptors. Passage 10 was the least inhibited by IgY (at least 10-fold), approximately 1000-fold more resistant to inhibition of infection than wild type. Infection by passages 8 and 13 was approximately 100-fold less inhibited by IgY than wild type. Passages 12 and 15 were as sensitive to inhibition of infection by IgY as the wild type CalX (Figure 2), while passages 11, 14, and 16 were moderately inhibited. The decrease in virus resistance to antibody neutralization among passages 10,11 and 12 also correlated with a corresponding decrease in virus yield. Among passages 10 to 16, a direct correlation between viral yield and inhibition of infection can be noted, where an increase in resistance to antibody resulted in high viral yield.

### HAI and antibody inhibition of infection do not correlate

Selected passages were also tested for HAI by IgY, presumably by sterically impeding the binding of virus to red blood cells. As seen with infectivity studies, all passages tested were inhibited by IgY at the level of HA activity. No passage tested was more than 8-fold more resistant to HAI by IgY than wild type (Figure 2), whereas some passages were more than 1000-fold resistant to antibody neutralization of infection. Passage 14 was the most resistant to HAI (8-fold greater than wild type) and was 10-fold less sensitive to neutralization of infection, suggesting a good correlation between HAI and neutralization of infection for this passage. Passages 8 and 13 showed only a four- and two-fold decrease, respectively, in HAI compared to wild type; but both were at least 100-fold more resistant to neutralization of infection than wild type. Passage 12 and 13 showed similar sensitivity to HAI, but 12 is inhibited about 10-fold more at the level of infection. Thus, resistance of a passage to inhibition of HA activity was a poor predictor of that passage's sensitivity to antibody inhibition at the level of infection, or of the overall viral yield.

### Diversity of plaque morphology suggests "boom" and "bust" cycles of individual subpopulations

**Figure 3:**
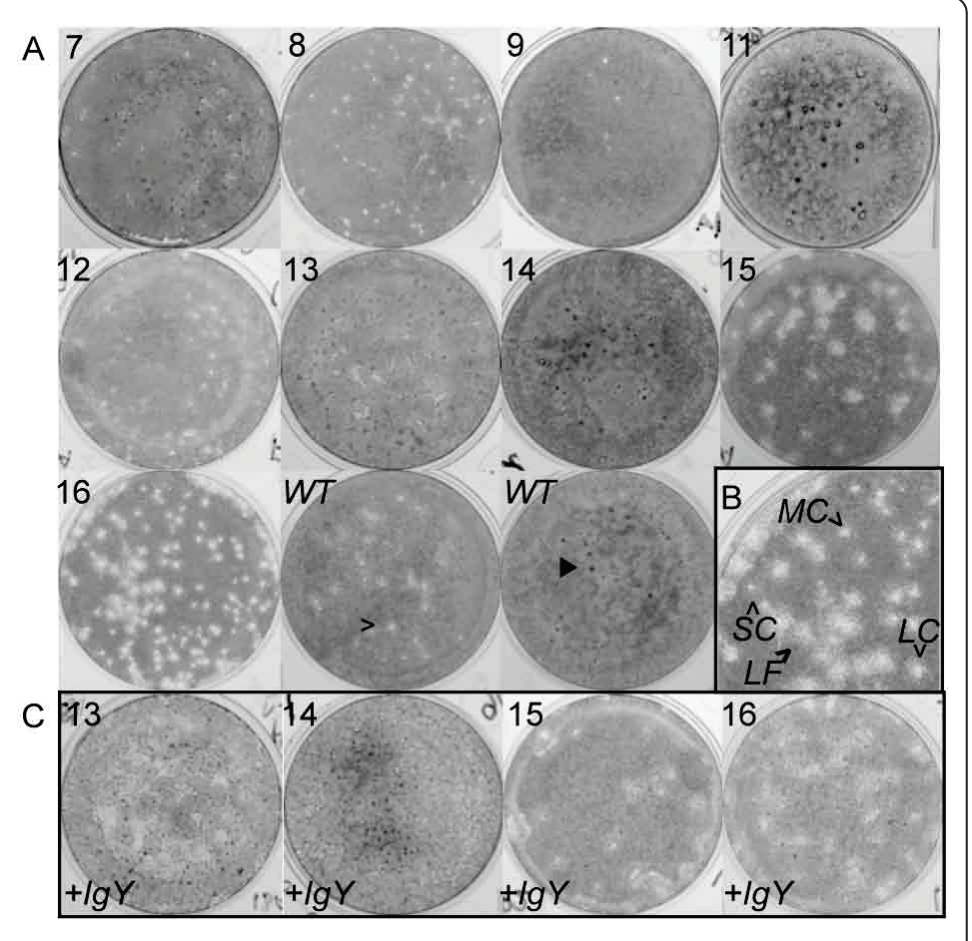
**Variation in plaque morphologies in IgY-selected viruses**. (A) Plaque assays performed in the absence of anti-CalX polyclonal chicken IgY antiserum on IgY-resistant isolates. Passage numbers are indicated in top left corner of each image. Note the presence of multiple plaque morphologies, including small clear plaques [open arrowhead] and dark spots [filled arrowhead] in wild type [*WT*] as well as IgY-resistant passages. (B) Enlargement of plaque assay of passage 16 showing plaques of small clear [*SC*], medium clear [*MC*], large clear [*LC*] and large fuzzy [*LF*] morphologies. (C) Plaque assays performed in the presence of 10^-3 ^diluted anti-CalX polyclonal chicken IgY antiserum. The variability of plaque morphologies suggests the presence of multiple variant lineages in most passages (see Additional file 1, Figure S1). Note that, except for passage 15, the constellation of different plaque types did not appreciably differ when infection took place in the presence of IgY, suggesting that no particular variant was significantly more resistant to antibody than others. Note that images were converted from color to grayscale, and brightness and contrast were adjusted to enhance plaque visibility. All image manipulations were performed in Adobe Photoshop.

Examination of plaques produced by the different passages allows us to infer additional phenotypic differences between viruses. Plaques observed varied enormously in both size and morphology (Figure 3). In early passages, most plaques were small or very small and had clear centers, although some larger plaques appeared "fuzzy", presumably due to incomplete lysis of cells. Dark, raised spots, which we presume to be knots of infected cells, were also apparent. We believe that each of these different plaque morphologies probably represents a distinct subpopulation of viruses, as described previously [28]. With the exception of the very large plaques observed in later passages, all of the plaque morphologies found in the IgY-selected passages were also observed in the wild type. Later passages show the presence of very large, fuzzy plaques, along with other plaques of a range of sizes, generally correlating with increase in infectious focus size (see below). It should be noted that there was a poor correlation between the plaque and focus formation assays for some passages, (e.g. passage 12, which showed a variety of focus sizes but only very small or medium/fuzzy plaques). Many wells contained a mixture of clear and fuzzy plaques, indicating that the differences in morphology were not merely the result of variations in experimental conditions.

To determine whether a particular plaque morphology might represent a subpopulation with increased resistance to antibody neutralization, plaque assays were conducted in the presence of IgY for passages 13-16. In general, all plaque morphologies were present when infection was performed in the presence of IgY, although all were reduced in number. An exception was passage 15, where the proportion of very large plaques was apparently higher in assays performed in the presence of IgY, suggesting the subpopulation represented by this particular plaque morphology was less sensitive to IgY inhibition than others. The virus titer from plaque assays conducted in the presence of IgY was similar to the virus yield obtained from liquid culture, again suggesting that cyclical "boom" and "bust" in virus yield is not due to large scale generation of defective interfering particles, since individual plaques presumably arise from a single initial infection. If there were large numbers of defective particles present in the inoculum, they would be able to inhibit infection in liquid culture, but not in the presence of the viscous CMC gel. The fluctuation in yield cannot be attributed to insufficient presence of IgY during generation of various passages, as the yield obtained reflects titer from the well where the ratio of IgY to virus would be expected to be the highest, in most cases higher than the ratio of IgY to wild type virus during the initial selection where the largest amount of input virus was used.

### Infectious focus size varies greatly, suggesting the presence of multiple cocirculating viral lineages

**Figure 4:**
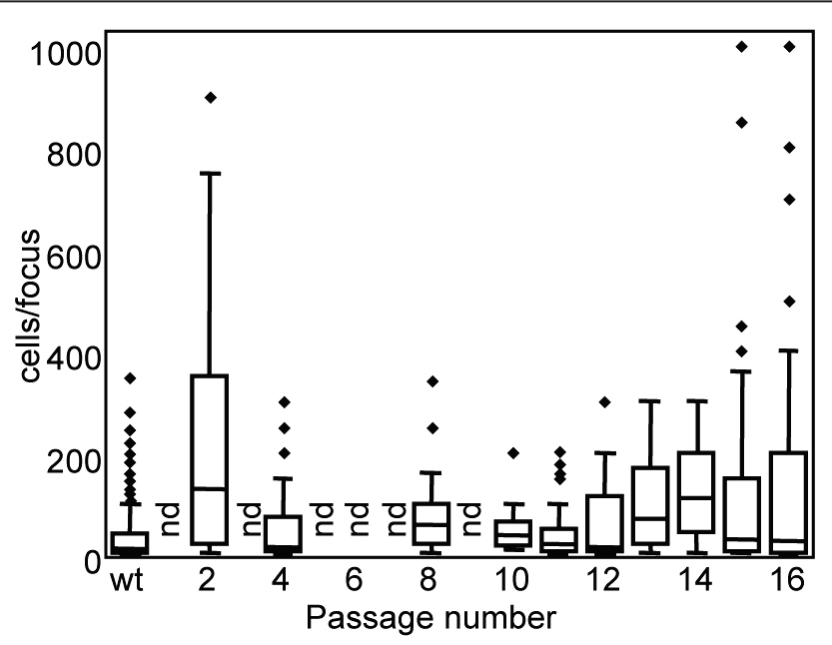
**Infectious focus size varies greatly from passage to passage**. Infectious foci of selected passages at limiting dilution were examined and the number of infected cells per focus were enumerated. (Median: horizontal line towards middle of box; 5^th ^and 95^th ^percentile values: lower and upper error bars, respectively; 25^th ^and 75^th ^percentile values: bottom and top of the box, respectively; outlier values: filled diamond; nd: not done). A minimum of 20 foci were examined for each passage. See Table 1 for pairwise statistical comparisons and numbers of foci analyzed. Data obtained for each passage were pooled from two to four independent experiments. Note that virus yield, degree of sensitivity of HA and infection to IgY, and focus size varied dramatically over the course of the study. Yield, antibody sensitivity, and focus size correlated poorly for individual passages.

To better understand differences between selected passages, cells were infected and overlaid with a CMC gel to permit the virus spread only between neighboring cells, rather than through the bulk liquid medium. Distinct individual foci fluorescently labeled with anti-influenza antibody were identified, and the number of fluorescent cells in each focus was enumerated (Figure 4). Focus size presumably reflects the rate of virus spread, which is probably affected by both the number of infectious particles shed by an infected cell (burst size) and the rate of viral replication. For most IgY resistant passages, the infectious foci were not uniform in size, suggesting the presence of multiple co-circulating viral lineages. The earliest passage tested, passage 2, yielded foci varying widely in size. Passage 2 had the largest median focus size and was significantly different from wild type (*p *< 0.001, Table 1). Focus size fluctuated from passage 4 (smallest median focus size) to passage 8, then further declined to passage 11 (small foci, least variable of all isolates tested, including wild type), suggesting that the populations giving rise to larger foci in the earlier passages had disappeared, presumably due to some sort of deleterious effect on fitness. Larger foci began to appear in passage 12, and foci in later passages were extremely variable, including some foci that were larger than any previously seen in this study. It should be noted that some variation in focus size was also observed for the wild type (Additional file 1, Figure S2), as expected since all RNA viruses are thought to exist as a complex population of related sequences [29,30]. Focus formation results were consistent in at least three replicate experiments for each passage tested, strongly suggesting that differences in focus size were not merely due to minor variations in experimental conditions such as initial cell density, overlay viscosity, and time to harvest.

**Table 1:** Pairwise Statistical Comparison^2 ^of Infected Focus Size^3^

passage	**Wt2 (10**^**-5**^**)**^**4**^ ***n *= 111**^**5**^	**16 (10**^**-5**^**)** *n *= 68	**15 (10**^**-3**^**)** *n *= 86	**14 (10**^**-5**^**)** *n *= 78	**13 (10**^**-5**^**)** *n *= 67	**12**^**6**^ *n *= 55	**11 (10**^**-3**^**)** *n *= 82	**10 (10**^**-4**^**)** *n *= 21	**8 (10**^**-5**^**)** *n *= 31	**4 (10**^**-5**^**)** *n *= 97
2 (10^-5^) *n *= 81	*p *< 0.001	*p *< 0.05	*p *< 0.01	ns	ns	*p *< 0.001	*p *< 0.001	ns	*p *> 0.05	*p *< 0.001
4 (10^-5^)	ns	ns	ns	*p *< 0.001	*p *< 0.01	ns	ns	ns	ns	
8 (10^-5^)	*p *< 0.05	ns	ns	ns	*p *> 0.05	ns	ns	ns		
10 (10^-4^)	ns	ns	ns	ns	ns	ns	ns			
11 (10^-3^)	ns	ns	ns	*p *< 0.001	*p *< 0.01	ns				
12^‡^	ns	ns	ns	*p *< 0.001	*p *< 0.05					
13 (10^-5^)	*p *< 0.001	ns	ns	ns						
14 (10^-5^)	*p *< 0.001	*p *< 0.01	*p *< 0.01							
15 (10^-3^)	*p *< 0.05	ns								
16 (10^-5^)	*p *< 0.05									

^2 ^*p *values were calculated using Kruskal-Wallis one-way analysis of variation (Prism V4.0, GraphPad Software, Inc).

^3 ^All infections performed in the absence of IgY.

^4 ^Highest dilution of isolate stock yielding infectious virus in the presence of IgY.

^5 ^Total number of foci examined.

^6 ^Combined data from 10^-4 ^and 10^-5 ^dilutions.

A comparison of foci between different dilutions of the same passage showed statistically significant differences in focus size (Additional file 1, Figure S1). For example, the median focus size for passage 14 was significantly smaller at the 10^-4 ^dilution of inoculum than at 10^-5^dilution, where most of the foci present were large. The reverse was true for passage 4, where larger foci were more evident at the lower dilution (10^-4^). This suggests that the different subpopulations present in these passages are not equally represented. Thus, for passage 14 at the higher dilution, there are fewer small foci compared to the large foci, since the subpopulation that gives rise to the small focus type has been diluted out. Similar discrepancies in focus size at different dilutions were seen with wild type. Both plaque and focus size are dependent on the rate of virus replication within the cell and the number of infectious particles released from each infected cell. The fact that differences in focus or plaque size and burst size do not necessarily correlate with calculated burst size suggest that there may be differences in rate of virus production in the infected cell for different passages.

Since we presume that the size of infectious foci is related to the rate of dissemination of progeny virus from cell to cell, we must assume that the larger foci result from infections that either progress much more rapidly or produce more infectious particles. We observe some differences in burst size (yield of particles), but this is not sufficient to explain the difference in focus size, suggesting that the rate of production of progeny viruses within the infected cell or the efficiency of spread between cells may also differ among the different populations. The avian polyclonal IgY appears to act in part by placing a severe restriction on the number of infectious particles in the population entering the cell and reproducing. Under these circumstances, those variants able to replicate the fastest or produce the largest number of progeny dominate the population at least transiently, as has been seen previously in other viruses [31]. Although we cannot exclude the possibility that the dynamics we observed were simply the result of viral genetic drift due to serial bottlenecks, the wild type stocks propagated during the course of study were also grown by limiting dilution but did not display a similar change in median focus size (Additional file 1, Figure S2), and the large plaques and foci seen with late passages with antibody were more prevalent than in wild type (Figure 3). Since our wild-type stocks are routinely grown at limiting dilution, they should be subject to bottlenecks of approximately the same size as our antibody-selected virus (0-9 infectious particles) at each passage. When the populations are compared based on the size of infectious foci, we observe statistically significant differences between antibody selected viruses and wild-type viruses passaged in the absence of antibody, suggesting that presence of antibody provides a different selective pressure than dilution alone, possibly by exerting selective pressure in multiple cycles of infection.

An influenza strain (NWS-Mvi) adapted to growth where cell surface receptors were drastically reduced by treatment with bacterial sialidase showed both an increase in viral transcription and a shift to fusion at a pH closer to neutral [11]. Interestingly, NWS-Mvi was also more prone to induce apoptosis in infected cells than its parent, suggesting that its robust replication has outstripped its ability to evade the host innate immune system. Data from both plaque and focus formation assays in this study shows the appearance of a population of very large infectious foci and plaques among late passages, suggesting the appearance of a highly robust variant within the population. Similarly, studies on the effect of combining mutations in an RNA virus, vesicular stomatitis virus (VSV), showed that two individually beneficial mutations when combined in the same genome nearly always produced a virus less fit than either single mutation alone, and two individually deleterious mutations combined in the same genome occasionally produced a double mutant virus more fit than either single mutant [32]. This antagonistic effect of beneficial mutations was observed at a much higher rate in VSV than in bacterial systems. Thus, even though the large plaque morphology would appear to have the advantage of producing more progeny or replicating more rapidly, it may do so at the expense of inducing death of the host cells before many produce viable viral progeny.

### Most HA sequences from IgY-selected passages are wild type

**Figure 5:**
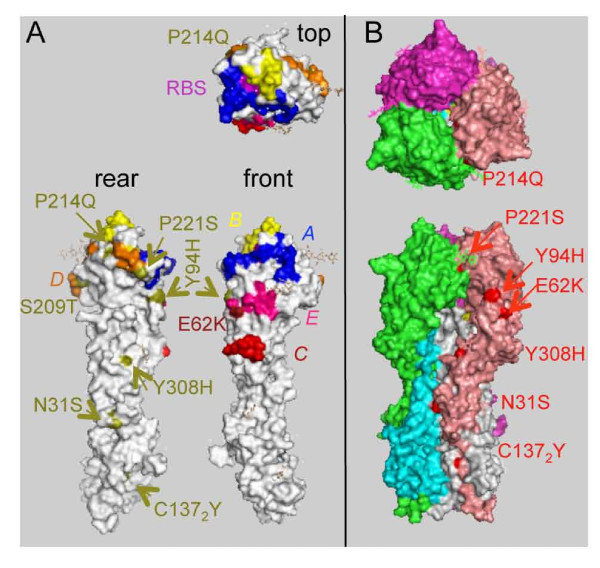
**Mutations in IgY-selected A/California/7/2004 HA**. **(A) **Amino acid residues found to be mutated in HA of IgY-selected viruses are indicated on the structure of the HA monomer viewed from the above (top), from the interior of the HA trimer (back), or from the front. The receptor binding site (RBS, magenta) and previously described human epitopes *A *(navy), *B *(yellow), *C *(pink), *D *(orange) and *E *(red) are shown [4]. Mutated residues observed in this study (Table 2) are highlighted (olive) and indicated by olive labels, except for E62K (dark red), falling in the previously described human epitope E. Note that we have not to date detected mutations in the *A *or *B *epitopes, most frequently associated with changes in HA in humans. **(B) **Observed mutations are shown in the context of the HA trimer. Individual monomers are colored salmon (HA1) and grey (HA2), green (HA1) and cyan (HA2), and purple (HA1; HA2 not visible). Mutations are shown in red, with red labels. Note that two mutations (Y94H and E62K) occur on the solvent-exposed surface near the trimer interface, although most (N31S, P214Q, P221S, Y308H, and C137_2_Y) are deeply buried within the interface and have the potential to alter intersubunit interactions. The figure was prepared using MacPyMol (http://www.pymol.org, DeLano Scientific LLC), using the crystal structure of A/X-31 (H3N2) HA ([49], PDB Accession ID: 2VIU) as a model.

Since our focus formation assay data suggest that different subpopulations are present in most of our IgY-selected passages, we cloned and sequenced individual HA genes from selected passages to characterize the diversity of the population. This would allow us to identify any HA mutations that might have increased the ability to infect in the presence of antibody (Table 2, Figure 5). Surprisingly, although some mutant sequences were found, the majority of the HA gene sequences obtained were identical to wild type. Of the mutants detected, coding changes (eight) outnumbered silent mutations (four), suggesting that the majority of the changes are likely to be authentically due to alterations in viral RNA, although we cannot definitively exclude the possibility that any particular mutation arose due to PCR amplification. Of the coding changes observed, five map in or near previously described antibody-neutralizing epitopes [33] (E62K, Y94H, P214Q, S209T, and P221S, see Figure 5. Note that the numbering system used is that for the mature, proteolytically processed HA molecule as in the crystal structure [34]. E62K is within the previously described "E" epitope, and Y94H is immediately adjacent to the "E" epitope on the edge of a large void separating two neighboring trimers. K62 was the prevalent residue in human H3N2 viruses prior to 1997. H94 has been found in human clinical isolates (e.g. A/New York/437/2000). Thus, mutations in these sites may be well tolerated, and even have antigenic significance. However, we do not see evidence of a particular mutant sequence being retained from passage to passage, suggesting that these changes alone are not sufficient to confer a selective advantage. Interestingly, none of the mutations we found were in the 'A' or 'B' epitopes previously described in H3HA, encompassing the residues most frequently associated with antigenic drift in human viruses [35]. The proportion of mutant sequences in the population varies over the course of the study, possibly suggesting the acquisition of a high frequency mutator phenotype in some passages (e.g. passage 11) [36].

**Table 2:** Mutations Observed in HA Genes from IgY-resistant Passages.

Passage	Mutant/total	**Amino acid changes**^**7 **^**[nucleotide changes]**^**8,9**^
2	2/12	P221S [C737T^10^]^11^
10	1/14	P214Q [C721A]
		N31S/silent [A140G/C1407T], Y94H/silent [T357C/T521C],
11^12^	4/9	S209T/silent/silent [G703C/G722A/A953G],
		Y308H/C137_2_Y [T999C/G1474A]
13^12^	1/2	E62K [G261A]
16	0/8	None

^7 ^Amino acids are numbered according to mature HA. Residues from the HA2 moiety are designated with the subscript "2" following the residue number.

^8 ^Nucleotide numbering is as for the full-length sequence of the mutant.

^9 ^Accession numbers will be supplied when sequence deposit is finalized.

^10 ^Mutations encoding amino acid changes are underlined.

^11 ^Both mutant isolates had identical sequence.

^12 ^Sequences obtained from PCR using *Taq *polymerase.

Several mutations map in regions of the molecule that might be expected to contribute to stabilizing interactions within the HA trimer (N31S, P221S) or intermolecular interactions within the mature HA monomer (Y308H, HA2 mutation C137_2_Y). Several of these (P214Q, P221S, and C137_2_Y) could be expected to radically alter the structure or stability of the HA monomer. P214Q and P221S affect residues that are invariant in available sequences of natural passages, and proline residues tend to be highly conserved due to the unique structural properties of this amino acid. P214 and P221 are invariant among human and avian H3 sequences currently available. N31S is buried within the structure, close to K27 and T28 in HA1 as well as R50 in the neighboring HA2. S31 has been observed in some human viruses (e.g. A/New York/335/1999).

The lone mutation detected in HA2, C137_2_Y, is buried. This mutation would disrupt a disulphide bond between the HA1 and HA2 chains, likely destabilizing the HA monomer, although this was found paired with Y308H, which may also affect interactions between HA1 and HA2. It is possible that changes that affect HA1/HA2 or inter-monomer interactions within the trimer might destabilize the neutral conformation of the HA, leading to a rise in the pH required for membrane fusion, as seen previously [11]. C137_2 _is invariant in all currently available human and avian H3 sequences, so the change we observe at this amino acid may simply represent a deleterious mutation that would be lost due to negative selection. Since the NA activity of CalX is very low both in previous reports [37] and in our hands, and other A/California/7/2004-like viruses have been shown to delete the NA segment on passage in MDCK cells [38], we feel it unlikely that NA antibodies play a significant role in selecting mutants in our system.

### Sequence of bulk PCR products obtained from viral RNA shows changes in replication genes

In order to begin to understand the basis of increased ability of our virus isolates to grow in the presence of neutralizing IgY, and to explain the changes is burst size and plaque morphology, we attempted to generate and sequence PCR products of all gene segments of our parental virus and two selected IgY-resistant passages, P2 and P16. We could successfully isolate PCR products from all gene segments except the NP gene segment of P16, which failed in repeated attempts despite the fact that all other gene segments were isolated from the same cDNA, and NP was successfully amplified from both the wild-type and P2 (see Additional file 1, Figure S2). This strongly suggests that the failure to amplify NP from P16 was not merely a technical issue, but may be related to changes in the sequences targeted by the PCR primers. Since these same sequences at the 5' and 3' ends are known to be involved in regulation of both transcription and replication (see [39] for review), there may be important alterations in the level of NP protein in cells infected with P16. Additionally, coding mutations were detected in PB2 (both P2 and P16) and NA (P16 only), along with silent mutations in PA (P16 only). We also observe reversion of non-coding changes in PB1 and PA found in CalX wt to the sequence found in A/Puerto Rico/8/34 (see Table 3). Mutations in PB2 have been associated with increased replicative fitness of human influenza viruses [2], presumably by altering viral transcription and replication, and have also been implicated in adaptation to novel hosts [40]. Changes in NA may alter virus spread [41] and interaction with the host innate immune system [42], and compensatory have been observed when HA is undergoing antigenic changes [43]. Since these particular mutations are apparently novel, the confirmation of any role in altering the phenotype of our antibody-resistant isolates would require the construction of reassortant viruses bearing each of these mutations individually in a wild-type background.

**Table 3:** Mutations observed in antibody-resistant isolates.

Gene [segment] (reference sequence, accession)	CalX wt Change: AA [NT]	P2 Change: AA [NT]	P16A1 Change: AA [NT]
PB2 [segment 1] (A/Puerto Rico/8/34, V00603.1)^13^	G309D [G953A], Y360S [A1106C], [C1540A], [G1815A]	G309D [G953A], Y360S [A1106C], [C1540A], [G1815A], **A661V [C2009T]**^**14**^	G309D [G953A], Y360S [A1106C], [C1540A], [G1815A], **A661V [C2009T], E676D [A2058C]**^**15**^
PB1 [segment 2] (A/Puerto Rico/8/34, J02151.1)	A53G [C282G], [T504A], S216G [A670G], ***[C730T]***^***16***^, L300F [G924C], H473L [A1442T], S517I [T1563G], [T1574G]	A53G [C282G], [T504A], S216G [A670G], L300F [G924C], H473L [A1442T], S517I [T1563G], [T1574G]	A53G [C282G], [T504A], S216G [A670G], L300F [G924C], H473L [A1442T], S517I [T1563G], [T1574G]
PA [segment 3] (A/Puerto Rico/8/34, V01106.1)^13^	K158R [A497G], ***K391E [A1194G]***, [T1308A]	K158R [A497G], [T1308A]	K158R [A497G], [**A783G]**, [T1308A]
HA [segment 4] (A/California/07/2004, EU103820.1)	100% homology	100% homology	100% homology
NP [segment 5] (A/Puerto Rico/8/34, J02147.1)^13^	[G306A], D247N [G784A]	[G306A], D247N [G784A]	Failed to amplify^17^
NA [segment 6] (A/California/07/2004, EU103978.1)	100% homology	100% homology	**V240I [G718A]**
M1 &M2 [segment 7] (A/Puerto Rico/8/34, V01099.1)	100% homology	100% homology	100% homology
NS1 & NS2/NEP [segment 8] (A/Puerto Rico/8/34, J02150.1)	100% homology	100% homology	100% homology

^13 ^CalX wt and antibody-selected isolates most closely related to A/reassortant/NIBRG-14(Viet Nam/1194/2004 × Puerto Rico/8/1934)(H5N1).

^14 ^Mutations present in P2 and/or P16 but not in wild type are shown in **bold **type

^15 ^Present as a mixture with wild type sequence at a mutant:wildtype ratio of approximately 1:2 (see Figure S3).

^16 ^Mutations present only in wild type are shown in ***bold italic ***type.

^17 ^See Figure S3.

## Conclusions

The host antibody response has an important role in the evolution of influenza in humans [5]. Changes in influenza A HA genes from year to year tend to occur most frequently in residues located on the solvent-exposed surface, and tend to cluster in regions also targeted by neutralizing monoclonal antibodies [4,5,44,45]. Despite the fact that birds and domestic poultry are thought to be important as both reservoirs and vectors for introducing novel influenza A viruses into the human population, little is known about forces that may shape the evolution of influenza in birds. To better understand this problem, we derived mutant influenza viruses capable of infecting MDCK cells in the presence of neutralizing polyclonal chicken IgY antiserum. During early passages, we saw a marked increase in the ability of our IgY-selected viruses to infect cells in the presence of antibody, suggesting IgY susceptible viruses were "weeded out" during the first rounds of selection, and the remaining viruses present in the population were relatively antibody resistant. The population of variants, based on infectious yield, burst size, focus size and plaque morphology, seems to be constantly changing, with one variant dominating transiently then being replaced by another, either because the "new" variant is more fit, or the previously dominant variant may have acquired a deleterious mutation.

The simplest explanation for acquisition of antibody-resistance would be that the resistant viruses had lost the ability to bind antibody due to the acquisition of mutations in antibody binding sites. If this were the case, we would expect such viruses to outcompete the viruses in the population retaining antibody binding, and we would expect inhibition by antibody to decrease as the population came to be dominated by viruses that could not bind antibody. However, when antibody-selected viruses were tested for the ability of antibody to inhibit HA activity *in vitro *and infection, we observed that the viruses were at least somewhat sensitive to antibody at both the level of hemagglutination and infection, even from passages that gave good yields when infection was carried out in the presence of antibody. Sequence data from multiple individual HA clones indicated that the majority of the HA sequences were identical to wild type, although some mutant sequences were detected. Some of the HA mutations observed mapped within or adjacent to previously described neutralizing epitopes areas of the HA. Other HA mapped in the intersubunit interface, the possibility that HA stability may have been affected. No individual mutant sequence was found in more than one passage. Changes were observed in both the PB2 and PA proteins between antibody-selected passages and wild type, suggesting the possibility that viral transcription or replication may be altered. After the initial "boom" passages, where apparently antibody-resistant viruses probably gained an advantage, several "bust" passages were observed where virus yield was extremely low. This cycle was repeated twice over the course of this study.

Studies of population dynamics in VSV showed that a mixed culture initiated with two clones of approximately equal fitness would eventually become dominated by one clone and the other would disappear, even if both clones experienced increases in fitness [46]. The "winner" presumably displaced the "loser by" acquiring mutations that conferred some vast superiority over the "loser". In our system, although we observe mutations that may occur in antigenic sites and therefore confer an advantage by ablating the binding of some antibodies in our polyclonal antiserum, these do not apparently confer sufficient advantage to allow viruses bearing these changes to outcompete wild type, so even if these changes do confer resistance to some antibodies, they are not the kind of "jackpot" mutations that would allow the viruses bearing them to displace others in the mixed population. Thus, our inability to select true antibody escape mutants using chicken polyclonal antiserum is in line with previous attempts to select dual escape mutants *in vitro *with murine anti-HA monoclonal antibodies, where escape mutants selected using a single monoclonal antibody occurred at a frequency of approximately one per 10^5 ^infectious doses [14,47], but the frequency of generating escape mutants was less than one in 10^9 ^per infectious dose when two or more monoclonal antibodies were combined [47]. This was interpreted as evidence that the epitopes "seen" by the murine monoclonal antibodies were distinct and independent. Our inability to select escape mutants using polyclonal chicken antiserum suggests that the chicken antibody response is also comprised of a mixture of high-affinity antibodies directed against multiple independent epitopes. In this light, it is interesting to note that viruses identified from year to year in the human population tend to differ by only a small number of HA changes, suggesting either that these changes confers a selective advantage in a large number of individuals in the population, in which case the antibody responses of different individuals might be expected to be very similar, or that factors other than HA changes may play an important role in the fitness of influenza viruses in the human population.

Our data demonstrate that, even within a simple model system, antigenic drift due to changes in HA is not the only mechanism by which viruses may gain a selective advantage, even in the presence of antibody. Evolutionary success is determined by multiple factors, including the ability to produce large numbers of progeny when the ability to bind and enter cells is restricted. Each viral gene may contribute to enhancing fitness, as seen during adaptation of influenza viruses to increased virulence in mice [48]. The "boom" and "bust" cycles we observe may reflect a situation where variant lineages rapidly come to dominate the population not solely because they are more competitive, but because other cocirculating populations have become less fit. Hence, general genetic and phenotypic characterization could provide critical information for selecting appropriate vaccine strains and predicting future influenza pandemics.

## Abbreviations

CMC: carboxymethylcellulose; DI: defective interfering; HA: hemagglutination or hemagglutinin; HAI: hemagglutination inhibition; MDCK: Madin-Darby canine kidney; m.o.i.: multiplicity of infection; NA: neuraminidase; p.i.: post-infection; VSV: vesicular stomatitis virus.

## Competing interests

The authors declare that they have no competing interests.

## Authors' contributions

RTR participated in study design, derived late passages of IgY-resistant virus, performed all assays described, generated and analyzed sequence data, and helped draft the manuscript. AR and EWN assisted with generating early passages of IgY-resistant virus. SNG and MEM produced anti-influenza antiserum. SJS conceived of the study, participated in its design, derived early passages of IgY-resistant virus and helped to draft the manuscript. All authors read and approved the final manuscript.

## Supplementary Material

Additional file 1 **Supplementary Information**. Table S1. Primers used in this study. Table S2. Accession numbers of reported sequences. Figure S1. Cocirculating variants are present at different concentrations. Figure S2. Differences in focus size of various wildtype preparations. Figure S3. Sequencing of complete genomes of wild-type, P2, and P16. Click here for file
