# Supplementary material for: "Boom" and "Bust" cycles in virus growth suggest multiple selective forces in influenza a evolution
Source: Virol J. 2011 Apr 18;8:180. doi: 10.1186/1743-422X-8-180 (PMC3339368; doi:10.1186/1743-422X-8-180)
Supplement: Additional file 1 — Supplementary Information. Table S1. Primers used in this study. Table S2. Accession numbers of reported sequences. Figure S1. Cocirculating variants are present at different concentrations. Figure S2. Differences in focus size of various wildtype preparations. Figure S3. Sequencing of complete genomes of wild-type, P2, and P16. [file 1743-422X-8-180-S1.PDF]

"Boom" and "Bust" Cycles in Virus Growth Suggest Multiple Selective Forces in Influenza A Evolution

SUPPLEMENTARY INFORMATION

Rajagowthamee R Thangavel[1], Aisha Reed[1] , Erin W Norcross[1], Sherrina N Dixon[1], Mary E Marquart[1], and Stephen J Stray[1,*]

[1] Department of Microbiology, University of Mississippi Medical Center, 2500 N State St, Jackson, MS 39216, USA
[*]Corresponding Author: Stephen Stray (email: sstray@umc.edu)

Table S1. Primers used in this study

number	name	sequence	Primer binding site
1	M13 FLUA 12MER[1]	TGTAAAACGACGGCCAGTAGCRAAAGCAGG	12
2	mod fluA uni[2]	GCGAGCAAAAGCAGG	12
3	PR8 PB2 FORWARD	AGCGAAAGCAGGTCAATTATATTC	24
4	PR8 PB2 REVERSE	AGTAGAAACAAGGTCGTTTT	2295
5	PR8 PB2 INT REV[3]	CTGAATCAATCTCCTGGTTGC	1156
6	PR8 PB2 INT FOR[4]	GACTTAGTTAGAGGACCAACG	1055
7	PR8 PB2 5' INT REV	CATCATCCATTTCATCCTA	162
8	PR8 PB2 3' INT FOR	GAACTGAGCAACCTTGCGA	2179
9	PR8 PB1 FORWARD	AGCGAAAGCAGGCAAACC	18
10	PR8 PB1 REVERSE	AGTAGAAACAAGGCATTTTTTCATG	2293
11	PR8 PB1 INT REV[5]	TGCAGTCCCCTCTATTAAGAGC	1206
12	PR8 PB1 INT FOR[6]	GGGTATATGTTTGAGAGCAAGAGT	1107
13	PR8 PB1 5' INT REV	TACTGATGTGTCCTGTTGAC	151
14	PR8 PB1 3' INT FOR	AGTATGGTGGAGGCTATG	2178
15	PR8 PA FORWARD	AGCGAAAGCAGGTACTGATC	20
16	PR8 PA REVERSE	AGTAGAAACAAGGTACTTTTTTGG	2210
17	PR8 PA INT REV[7]	GTGCCATGTTCTCACCAAGTGCCCA	1127
18	PR8 PA INT FOR[8]	ACATTGAGAATGAGGAGAAAATTC	1087
19	PR8 PA 5' INT REV	TCTGAATACATGAAGCATAC	154
20	PR8 PA 3' INT FOR	GACAACCTTGAACCTGGGA	2062
21	H3 HA M13 FOR[9]	TGTAAAACGACGGCCAGTAGCAAAAGCAGGGGATAATTC	21
22	H3 HA M13 REV[9]	CAGGAAACAGCTATGACCAGTAGAAACAAGGGTGTTTT	1720
23	Bm-HA-1[10]	TATTCGTCTCAGGGAGCAAAAGCAGGG	13
24	HA 5' INT REV	TCAGTAGCATTAGTAACT	167
25	HA 3' INT FOR	ATGATGTATACAGAGATGA	1558
26	HA2 INT REV	CTCAACATATTTCTCGA	1303
27	CAL/07/04 NA M13 FOR	TGTAAAACGACGGCCAGTATGAATCCAAATCAAAAGAT	40
28	CAL/07/04 NA M13 REV	CAGGAAACAGCTATGACCGAAAGCTTATATAGGCATGAG	1399
29	NA 5' INT REV	TACACTATCTCTGTTATGT	185
30	NA 3' INT FOR	GCAAAAGCTGCATCAATCG	1262
31	PR8 NP FORWARD	AGCAAAAGCAGGGTAGAT	18
32	PR8 NP REVERSE	AGTAGAAACAAGGGTATTTTTC	1499

Table S1, continued

number	name	sequence	Primer binding site
33	PR8 NP 5' INT REV	GTGCACATTTGGATGTAGAAT	144
34	PR8 NP 3' INT FOR	GTGCAAGACCAGAAGATGTG	1401
35	PR8 M FORWARD	AGCGAAAGCAGGTAGATATTG	21
36	PR8 M REVERSE	AGTAGAAACAAGGTAGTTTTTTAC	1004
37	PR8 M 5' INT REV	GGATTGGTCTTGTCTTTAG	136
38	PR8 M 3' INT FOR	GCACTTGACATTGTGGATTC	828
39	BM-NS-1[10]	TATTCGTCTCAGGGAGCAAAAGCAGGGTG	15
40	BM-NS-890R[10, 11]	ATATCGTCTCGTATTAGTAGAAACAAGGGTGTTTT	890
41	PR8 NS 5' INT REV	GATGTCCAGACCGAGAGTACT	156
42	PR8 NS 3' INT FOR	GGTTGATTGAAGAAGTGAGA	738

1 M13-tagged influenza A universal primer
2 First three nucleotides (GCG) added at 5’ end to increase Tm for PCR and for ease of synthesis
3 Used with #3 to generate subgenomic amplicon for sequencing.
4 Used with #4 to generate subgenomic amplicon for sequencing.
5 Used with #9 to generate subgenomic amplicon for sequencing.
6 Used with #10 to generate subgenomic amplicon for sequencing.
7 Used with #15 to generate subgenomic amplicon for sequencing.
8 Used with #16 to generate subgenomic amplicon for sequencing.
9 From Ghedin E*, et al.* (2005) Large-scale sequencing of human influenza reveals the dynamic nature of viral genome evolution. *Nature* 437:1162-1166.
10 From Hoffmann E, Stech J, Guan Y, Webster R, & Perez D (2001) Universal primer set for the full-length amplification of all influenza A viruses. *Archives of Virology* 146:2275–2289.
11 Also used with Bm-HA-1 to amplify HA

Table S2. Accession numbers of reported sequences.

Gene (mutation)	Source	Sequence name	Accession
HA (P221S)	P2 (clone)	Cal04IgYP2HA_P221S_S12C6	JF690273
HA (P221S)	P2 (clone)	Cal04IgYP2HA_P221S_S12C7	JF690274
HA (P215Q)	P10 (clone)	Cal04IgYP10AHA_P215Q	JF690275
HA (Y94H)	P11 (clone)	Cal04IgYP11AHA_Y94H	JF690276
HA (Y308H/C137Y1)	P11 (clone)	Cal04IgYP11AHA_Y308H/C137Y	JF690272
HA (S209T)	P11 (clone)	Cal04IgYP11AHA_S209T	JF690277
HA (E62K)	P13 (clone)	Cal04IgYP13AHA_E62K	JF690278
HA (wt)	P16 (clone)	Cal04IgYP16AHA_C10	JF690279
PB2	Wt (bulk PCR)	Cal04XPR8PB2	JF690264
PB1	Wt (bulk PCR)	Cal04XPR8PB1	JF690265
PA	Wt (bulk PCR)	Cal04XPR8PA	JF690266
HA	Wt (bulk PCR)	Cal04XPR8HA	JF690267
NP	Wt (bulk PCR)	Cal04XPR8NP	JF690268
NA	Wt (bulk PCR)	Cal04XPR8NA	JF690269
M	Wt (bulk PCR)	Cal04XPR8M	JF690270
NS	Wt (bulk PCR)	Cal04XPR8NS	JF690271
PB2	P2 (bulk PCR)	CALXIGYP2PB2	JF690248
PB1	P2 (bulk PCR)	CALXIGYP2PB1	JF690249
PA	P2 (bulk PCR)	CALXIGYP2PA	JF690250
HA	P2 (bulk PCR)	CALXIGYP2HA	JF690251
NP	P2 (bulk PCR)	CALXIGYP2NP	JF690252
NA	P2 (bulk PCR)	CALXIGYP2NA	JF690253
M	P2 (bulk PCR)	CALXIGYP2M	JF690254
NS	P2 (bulk PCR)	CALXIGYP2NS	JF690255
PB2	P16 (bulk PCR)	CalXIgYp16A1PB2	JF690256
PB2 (E676D)	P16 (bulk PCR)	CalXIgYp16A1PB2E676D	JF690257
PB1	P16 (bulk PCR)	CalXIgYp16A1PB1	JF690258
PA	P16 (bulk PCR)	CalXIgYp16A1PA	JF690259
HA	P16 (bulk PCR)	CalXIgYp16A1HA	JF690260
NA	P16 (bulk PCR)	CalXIgYp16A1NA	JF690261
M	P16 (bulk PCR)	CalXIgYp16A1M	JF690262
NS	P16 (bulk PCR)	CalXIgYp16A1NS	JF690263

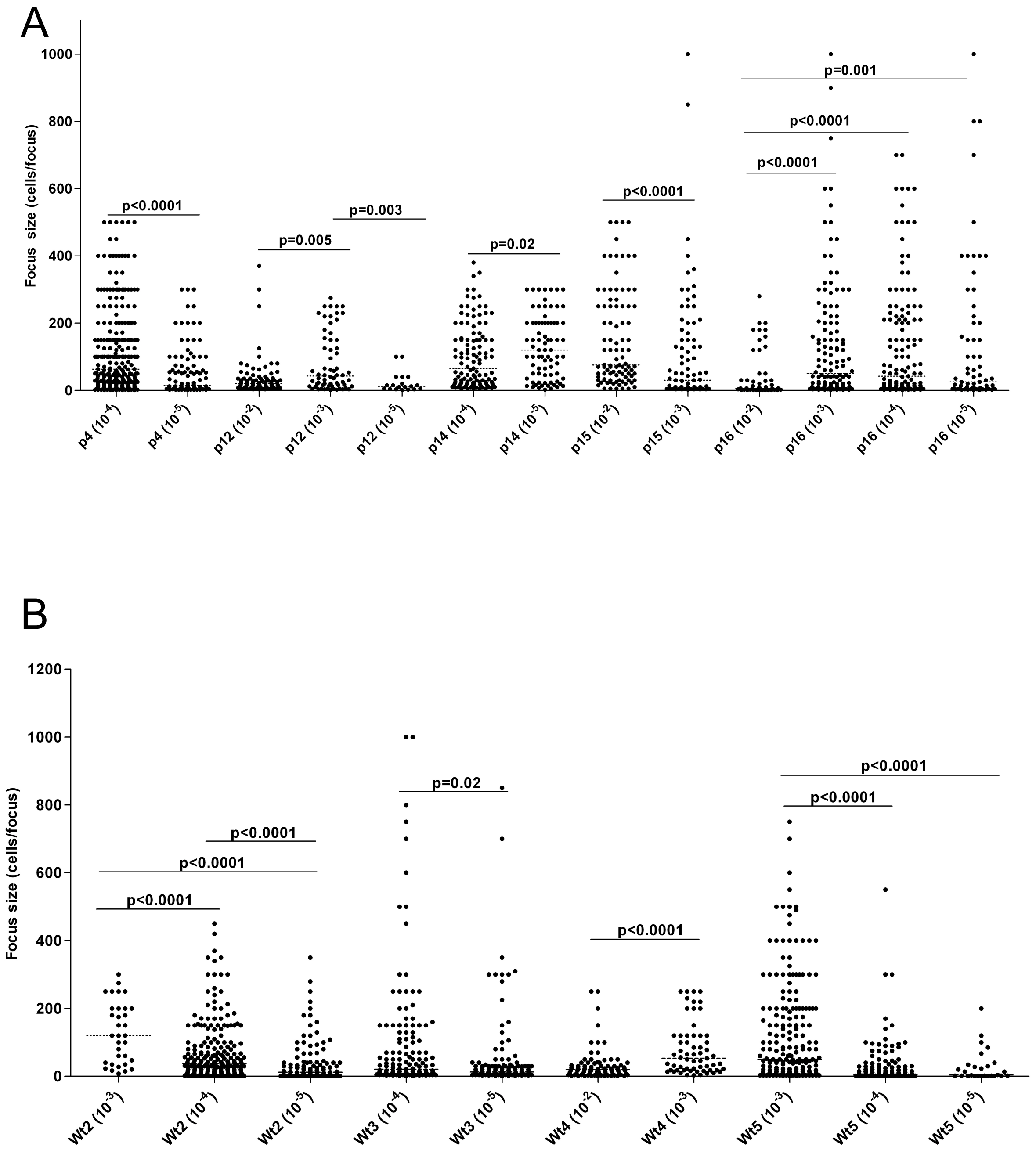

Fig. S1. Cocirculating variants are present at different concentrations.
Infectious foci in different dilutions of IgY-resistant passages (*A*), and wildtype preps (*B*). Data are combined from 2 to 3 experimental replicates (at least 20 foci per passage). Dotted horizontal lines represent median values for each population. Pairwise statistical comparisons were performed using Mann-Whitney non-parametric *U* test.

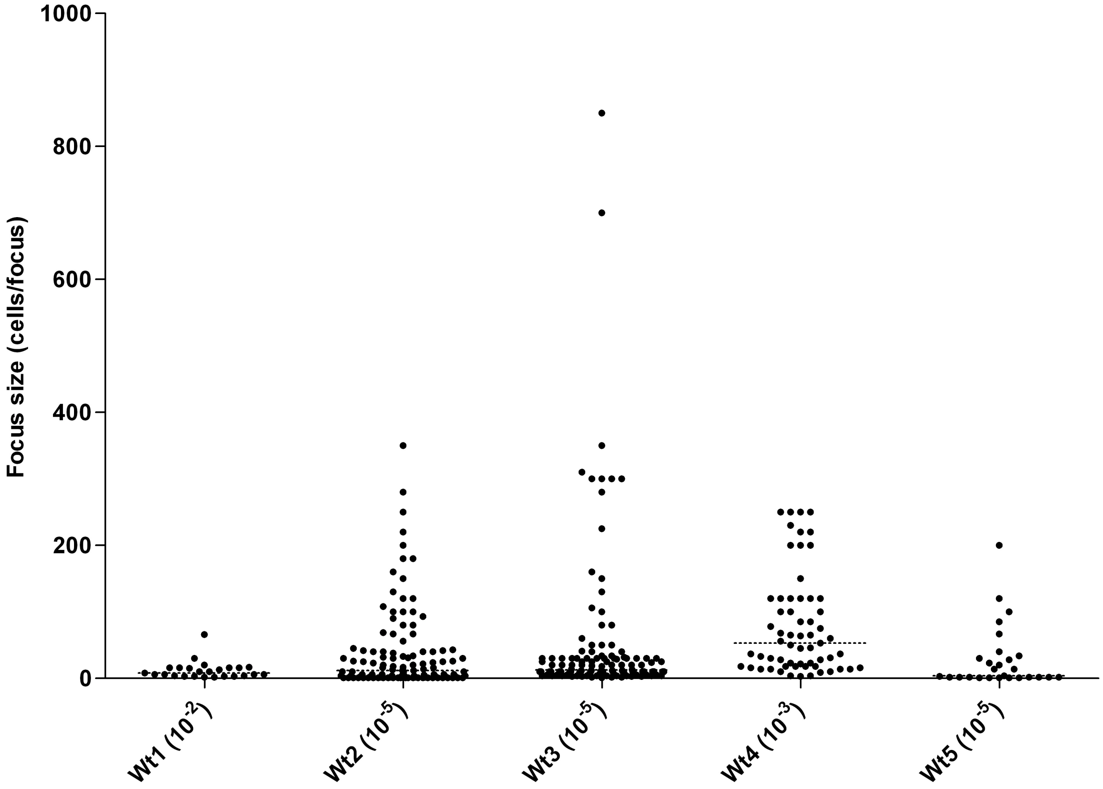

Fig. S2: Differences in focus size of various wildtype preparations.
Preparations of CalX grown in our laboratory at various times were compared by focus formation assay. Note that one prep, Wt4, produced infectious foci that were significantly larger than others (p<0.05), for reasons that are unclear to us. Data are combined from 2 to 4 experimental replicates (at least 20 foci per passage). Dotted horizontal lines represent median values for each population.

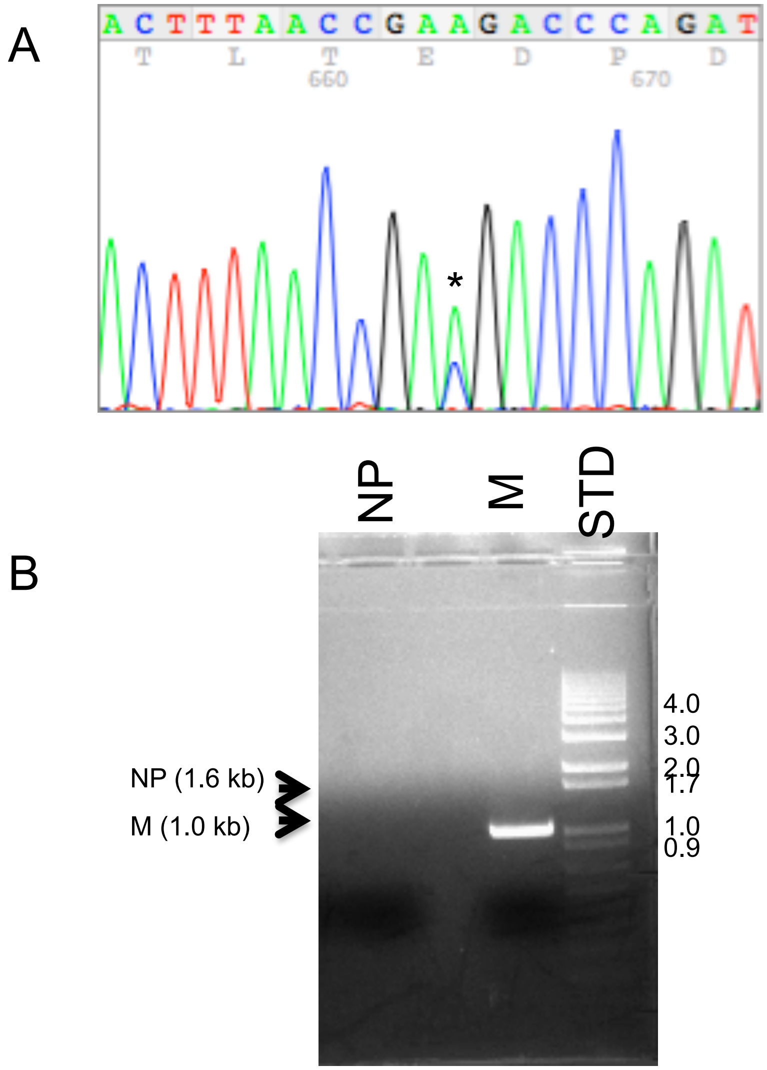

Fig. S3. Sequencing of complete genomes of Wild-type, P2 and P16 (A) Sequencing of bulk PCR of p16 shows evidence of a mixture of genomic sequences (asterisk). The majority sequence is identical to PB2 of P2, while the minority sequence represents the mutation E676D (see Table 3) (B) NP gene of p16 failed to amplify product. The same primers were used to successfully amplify sequence from the NP gene segment (segment 5, lane "NP") of cDNA prepared from wild-type and p2 isolates (not shown), and the same cDNA preparation was used to successfully generate amplification product with PCR primers specific for segment 7 (lane "M") and the remaining 7 genes. Image shown is representative of at least three attempts. Primers are described in Table S1.
